# Advancing quality culture in health professions education: experiences and perspectives of educational leaders

**DOI:** 10.1007/s10459-020-09996-5

**Published:** 2020-10-12

**Authors:** G. W. G. Bendermacher, D. H. J. M. Dolmans, W. S. de Grave, I. H. A. P. Wolfhagen, M. G. A. oude Egbrink

**Affiliations:** 1 Faculty of Health, Medicine and Life Sciences, Institute for Education – Department of Strategy and Policy, School of Health Professions Education, Maastricht University, PO Box 616, 6200 MD Maastricht, The Netherlands; 2 Faculty of Health, Medicine and Life Sciences, Department of Educational Development and Research, School of Health Professions Education, Maastricht University, Maastricht, The Netherlands; 3 Faculty of Health, Medicine and Life Sciences, Institute for Education and Department of Physiology, School of Health Professions Education, Maastricht University, Maastricht, The Netherlands

**Keywords:** Appreciative inquiry, Continuous quality improvement, Educational leadership, Faculty development, Organisational learning, Quality culture

## Abstract

The concept of quality culture has gained increased attention in health professions education, drawing on insights that quality management processes and positive work-related attitudes of staff in synergy lead to continuous improvement. However, the directions that guide institutions from quality culture theory to educational practice have been missing so far. A prospective qualitative case study of three health professions education programmes was conducted to explore how a quality culture can be enhanced according to the experiences and perspectives of educational leaders. The data collection was structured by an appreciative inquiry approach, supported with vignette-based interviews. A total of 25 participants (a selection of course coordinators, bachelor coordinators and directors of education) reflected on quality culture themes to learn about the best of what is (Discover), envision positive future developments (Dream), identify actions to reach the desired future (Design), and determine how to support and sustain improvement actions (Destiny) within their own educational setting. The results are presented as themes subsumed under these four phases. The experiences and perspectives of educational leaders reveal that peer learning in teams and communities, attention to professional development, and embedding support- and innovation networks, are at the heart of quality culture enhancement. An emphasis on human resources, (inter)relations and contextual awareness of leaders stood out as quality culture catalysts. Educational leaders are therefore encouraged to especially fuel their networking, communication, coalition building, and reflection competencies.

## Introduction

Many schools for health professions education (HPE) nowadays face the challenges of increasing student numbers, rapidly evolving educational approaches and technologies, a volatile financial environment and increased accountability pressures (Frenk et al. 2010). These circumstances call for an amplified focus on institutions’ continuous improvement capacities (Blouin and Tekian 2018). Since the 1990′s, educational quality enhancement methods have been carried forward by waves of research on organisational culture and performance, total quality management, and an integration of both into the more topical concept of ‘quality culture’ (Harvey and Williams 2010; EUA 2012). The latter concept is commonly used in higher education and originates from insights that quality management and control approaches are too technocratic and top-down and therefore fail or even have adverse effects (Newton 2000; Lomas 2004). There is a growing consensus that an organisational culture for quality requires a more holistic approach in which quality systems and instruments, competencies, and individual as well as collective values are not seen as separate entities but are combined into one overarching concept—the concept of quality culture (Ehlers 2009).

### Defining quality culture

The most often used definition of quality culture has been formulated by the European University Association (EUA). The EUA states that a quality culture is:

an organisational culture that intends to enhance quality permanently and is characterised by two distinct elements: on the one hand, a cultural/psychological element of shared values, beliefs, expectations and commitment towards quality and on the other hand, a structural/managerial element with defined processes that enhance quality and aim at coordinating individual efforts (EUA 2006, p. 10).

Rather than emphasising control, assurance and compliance to standards, a quality culture focuses on change, development and innovation (Ehlers 2009). Positive psychological attitudes of staff (e.g. ownership and commitment) are considered working mechanisms of a quality culture, since they reinforce staff contributions to continuous improvement processes. The notion of quality culture is often captured in one breath with strategies that emphasise collaboration, shared goals and visions, stakeholder involvement, and a supportive communication climate (Bendermacher et al. 2017).

Quality cultures in HPE institutions seem to maintain an emphasis on stability and formal procedures, whereas cultures characterised by cohesiveness and motivation might be more open to engage in reflections on continuous quality improvement (Blouin 2019; Blouin et al. 2019; Ovseiko and Buchan 2012). Pololi et al. (2009) found that the values of academic staff might not be in tune with organisational values in medical education and that this is expressed in an undervaluing of tasks performed in education. Efforts to advance the quality culture in HPE institutions are also found to be driven by attempts to promote internal integration, and at the same time be better prepared for external (accreditation) demands (Al-Shehri and Al-Alwan 2013; Blouin and Tekian 2018; Bendermacher et al. 2020). HPE leaders can positively contribute to building a common set of quality related values (Bland and Wersal 2002). Through sharing values and involving academic staff members in goal setting, their commitment to education can be reinforced. This is important, since in this way, a better balance could be reached between teaching roles and roles that academics also have in research and/or healthcare (Cantillon et al. 2016).

### A further exploration of leadership in health professions education

Previous studies have contributed to an increased insight in good leadership practices and desired leadership skills. For instance, Lieff and Albert (2012) concluded that medical education leaders navigate between intrapersonal (e.g. creating self-awareness), interpersonal (e.g. community building), organisational (e.g. task and goal setting) and managerial (e.g. strategy development) fields of practice. In a study by Bordage et al. (2000), being visionary, open-minded, and trustworthy stood out as preferred personality attributes of HPE leaders. Contemporary research implies that the strategic, employee-oriented and value driven processes associated with the influence of leaders do not merely depend on powerful, charismatic, or visionary leadership (Sandhu 2019). Rather, a shift is taking place from leader-centred theories to understanding leadership impact based on shared, collaborative, and distributed approaches (McKimm and Lieff 2013). There is increasing recognition that situational (or adaptive) leadership is required to address the complexity inherent in the academic environment (Velthuis et al. 2018). As leadership in HPE is multi-layered, within the reality of (medical) school hierarchies, one might be a leader and a follower at the same time (McKimm and O’Sullivan 2016). Moreover, instead of being mere leadership recipients, academics can steer the leadership of others and impact organisational developments through co-creation (Uhl-Bien 2014).

### Research gaps

A main challenge ahead for HPE institutions and their leaders lies in finding the stepping stones that pave the path from quality culture theory to organisational practice (Kottman et al. 2016). Despite the importance attributed to quality culture and educational leadership in their own right, insights into how leaders can contribute to quality culture enhancement remain un(der)recognised by some and are either unclear or misunderstood by a good many (Scott et al. 2008; Bryman 2007; Lieff and Yammarino 2017). This lack of insight might result from a scarcity of research that takes contexts into account when studying the phenomena of quality culture or leadership (Mohelska and Sokolova 2014; Harvey and Stensaker 2008; Uhl-Bien 2006; Hallinger 2018). Moreover, while quality culture studies often include a strong theoretical component, many studies on educational leadership lack reference to supporting theory (Bendermacher et al 2017; Dopson et al. 2019). A third gap in the literature concerns the fact that a majority of leadership studies concentrate on the highest management level (Bolden et al. 2012).

### Research aim

This study aimed to bridge the quality culture theory–practice gap by addressing the question: *How can a quality culture in health professions education be enhanced according to the experiences and perspectives of educational leaders?* We are especially interested in how HPE leaders working at different management levels help shape a quality culture and what their preferences for further (organisational and educational) development are. We focussed on leaders as central actors influencing culture, although it should be acknowledged that leaders are influenced by subcultures themselves as well (Bland et al. 2000).

## Methods

### Design

We followed the principles of a prospective qualitative case design to explore educational leader’s experiences and perspectives on the advancement of a quality culture in the setting of their own organisation. This design encompassed a deductive testing of hypothetical ‘ideal’ situations against data gathered from three undergraduate (bachelor) programmes in HPE (Bitektine 2008). Vignette-based interviews were used to let educational leaders reflect on professional dilemmas and to invite them to identify opportunities for further organisational development (Bernabeao et al. 2013). Our instrumental approach to the case analysis focused on learning from similarities and redundancy across three cases (rather than on identified variety within or across cases) (Stake 2008).

### Study approach

Our study approach followed the theoretical and methodological ideas behind ‘appreciative inquiry’. Grounded examples from the organisation’s positive past are first identified and subsequently used to portray images of the organisation’s positive future (Cooperrider and Whitney 1999; Sandars and Murdoch-Eaton 2017). The appreciative inquiry approach entails an exploration of four interconnected phases that together can be used to initiate and guide organisational change: ***Discovery*** (learn about ‘the best of what is’), ***Dream*** (envision possibilities for the desired future), ***Design*** (identify actions to reach the desired future), and ***Destiny*** (determine how to support and sustain future development actions) (Stavros et al. 2015).

### Study setting and participants

This study was conducted from November to December 2019, at Maastricht University’s Faculty of Health Medicine and Life Sciences (FHML), situated in The Netherlands. All programmes at FHML apply Problem-Based Learning (PBL). Three directors of education bear responsibility for education in the FHML domains ‘Health’, ‘Medicine’, and ‘Biomedical Sciences’; each director is responsible for multiple bachelor/master programmes. Three FHML bachelor programmes, each led by a bachelor coordinator, made up the cases: (1) the bachelor in Biomedical Sciences with 336 first-year students who started in 2019, (2) the bachelor in Health Sciences with 285 students who started in 2019, and (3) the bachelor in Medicine, with 321 students who started in 2019. The programmes are structured in 4–10-week thematic courses (each programme includes 25–35 courses). The courses are designed, implemented, and evaluated by planning groups consisting of 3–6 staff members from a variety of academic disciplines and are headed by a course coordinator. Most planning groups also include one or two student members.

#### Participant selection and inclusion

Our study focused on educational leadership at course, bachelor and domain level. We purposefully invited course coordinators from each year 1, 2 and 3 of the programmes to participate in an individual interview. We approached those course coordinators with the highest overall average ratings for the student course evaluation items about the ‘learning effect’, ‘quality of organisation’, ‘quality of learning activities’, and ‘alignment of different parts of the course’ in the 2017–2018 and 2018–2019 academic years. In total, we invited 6 coordinators from year 1. Furthermore, 10 coordinators from year 2 were invited since the curricula then offer more elective/specialisation courses. For year 3, 3 coordinators were invited since the curricula then include less obligatory courses and/or a thesis period. In addition to the 19 interviews with course coordinators (N = 6 or N = 7 per programme), the bachelor coordinators (N = 3) and directors of education (N = 3) were interviewed, resulting in a total number of 25 interviews; 9 female (36%); 16 male (64%). The participants were affiliated to 15 different departments. Table 1 provides further information on the participants.

**Table 1:** Background information on study participants

Management level	Course	Bachelor	Domain ^a^	Total	
	N	N	N	N	%
*Participant data*					
Female	6	2	1	9	36
Male	13	1	2	16	64
Total	19	3	3	25	100
*Position*					
Full Professor	4	2	2	8	32
Associate Professor	6	1	1	8	32
Assistant Professor	4	0	0	4	16
Lecturer	5	0	0	5	20
*Educational background*^*b*^					
Biomedical/Basic sciences	9	2	1	12	48
Social sciences	7	1	1	9	36
Medicine	3	0	1	4	16

Work experience	M	M	M	M	range
Years in role	3	5	5	3	1–6
Years at institution	25	20	29	25	11–39
Represented Departments					
Anatomy and Embryology, Clinical Psychology, Epidemiology, Genetics and Cell Biology, Gynaecology and Obstetrics, Health Promotion, Health Services Research, Human Biology, Medical Microbiology, Nutrition and Movement Sciences, Orthopaedics, Pathology, Psychiatry and Neuropsychology, Skills lab, Social Medicine					

*N* Number of participants, *M* mean

^a^A domain covers multiple bachelor/master programmes

^b^Refers to undergraduate education of interviewees (basic/biomedical sciences e.g. refers to pharmacology, chemistry; social sciences e.g. refers to sociology, health Sciences)

## Research team and reflexivity

The first author (GB) is an educational policy advisor who is also working as a part-time PhD candidate. He has cooperated with several of the interviewed staff members in quality management procedures. WG was responsible for interviewing all participants. He is an educational psychologist with a broad expertise in faculty development. WG took on the interviewing task not only because of his qualitative research experience, but also because of his relatively independent relation to the participants and programmes under study. In so doing, we intended to contribute to an open character of the interviews. The research team further consisted of two educational scientists with a focus on quality assurance (IW and DD), and a medical physiologist working as professor of medical education (ME). Two team members (IW and ME) hold senior management positions at the FHML Institute for Education (as vice-director and scientific director, respectively). We are aware that the experiences derived from our different roles and positions have shaped our perspectives. The diversity in our backgrounds however also helped us to broadly reflect on the data.

### Creation of vignettes and interview guide

All participants were interviewed with the use of five vignettes. These vignettes were designed by the first author (and thereafter reviewed and approved by the other team members) based on previous quality culture research in medical schools (Bendermacher et al. 2020). For four of the vignettes, a card that summarised the main concepts was provided. In the fifth vignette, main concepts were marked in bold. An interview guide developed by GB and WG supported the discussion of the vignettes (see “Appendix”). This guide was structured according to the “4-D” phases of appreciative inquiry. The interview guide also included a sensitising opening question on the study programme culture. To prevent social desirability bias from affecting results, we did not ask course coordinators to evaluate higher management in their own educational domain or vice versa. The vignettes and interview guide were tested by GB and WG in interviews with a former director of education and a former bachelor and course coordinator of FHML’s Medicine programme. No changes to the vignettes were deemed required after the pilot interview sessions. Changes made to the interview guide were the incorporation of an open question on personal leadership style and an integration of the questions on the design and delivery stages of appreciative inquiry.

### Data collection and management

All interviews (average duration 73) were held in Dutch. The audio-taped interviews were transcribed verbatim and pseudonymised by GB. The audio files and interview transcripts were stored on GB’s secured personal research drive (only accessible by the data manager of Maastricht University’s School of Health Professions Education). To check for the adequacy of transcripts, all participants were asked to review these and provide their approval. After the participant’s approval of the transcripts, the audio recordings were deleted. Participants during the transcript review stage could inform GB should they want to omit, alter or further explain transcript parts. Such changes or additions were made for five participants. An authorised translator ensured the quality of the selected quote translations from Dutch to English.

### Data analysis

**Fig. 1:**
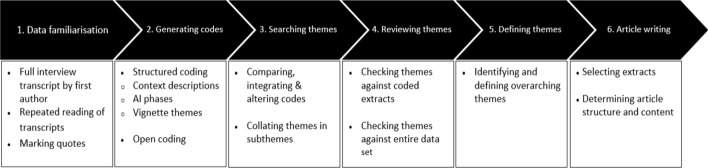
Six phases of thematic analysis. Based on Braun And Clarke (2006)

Data were analysed via the six steps of thematic analysis as described by Braun and Clarke (2006) (see Fig. 1). In the first step, GB transcribed the audio files in order to gain familiarity with the data. Please note that all interviewees have provided their written consent for GB’s direct access to audio files. To ensure further anonymity, other team members did not have access to the audio files. GB and WG repeatedly read transcripts and marked salient quotes: a process already initiated during the data collection (simultaneous interviewing and transcribing) phase, in order for WG and GB to be able to discuss their first impressions. In the second step (code generation), GB and WG independently coded all interviews by means of a combined structured and open coding approach. The structured approach included coding of illustrative context examples per case (study programme), appreciative inquiry phases, and vignette themes. Simultaneous open coding allowed for a broader interpretation of the data (Saldana 2013). In the third step (theme search), GB and WG met to discuss, merge, and refine codes and identify subthemes. Subsequently, in the fourth step (theme review), the outcomes of the previous steps were shared with the full research team. The team members that had not been involved in the previous steps reviewed the selection of anonymised salient quotes that were clustered per case (a total of 186 quotes were reviewed). The subthemes identified by GB and WG were checked against these extracts and further refined based on group discussions. GB then checked whether the identified subthemes adequately covered the entire dataset. In the fifth step (defining themes), the team identified themes that overarched the cases based on subtheme agglomeration and cross-case comparisons. We did not identify particular subthemes that stood out in only one of the cases. Through several team discussions, we reached consensus on the interpretation of findings. In the sixth and final step (article writing), the team members contributed to the manuscript in various iterations. The analysis was supported by ATLAS.ti software application (version 8.3, GmbH, Berlin, Germany).

### Ethical considerations

This study was approved by Maastricht University’s Health Medicine and Life Sciences Ethical Review Committee (FHML-REC/2019/042).

## Results

**Fig. 2:**
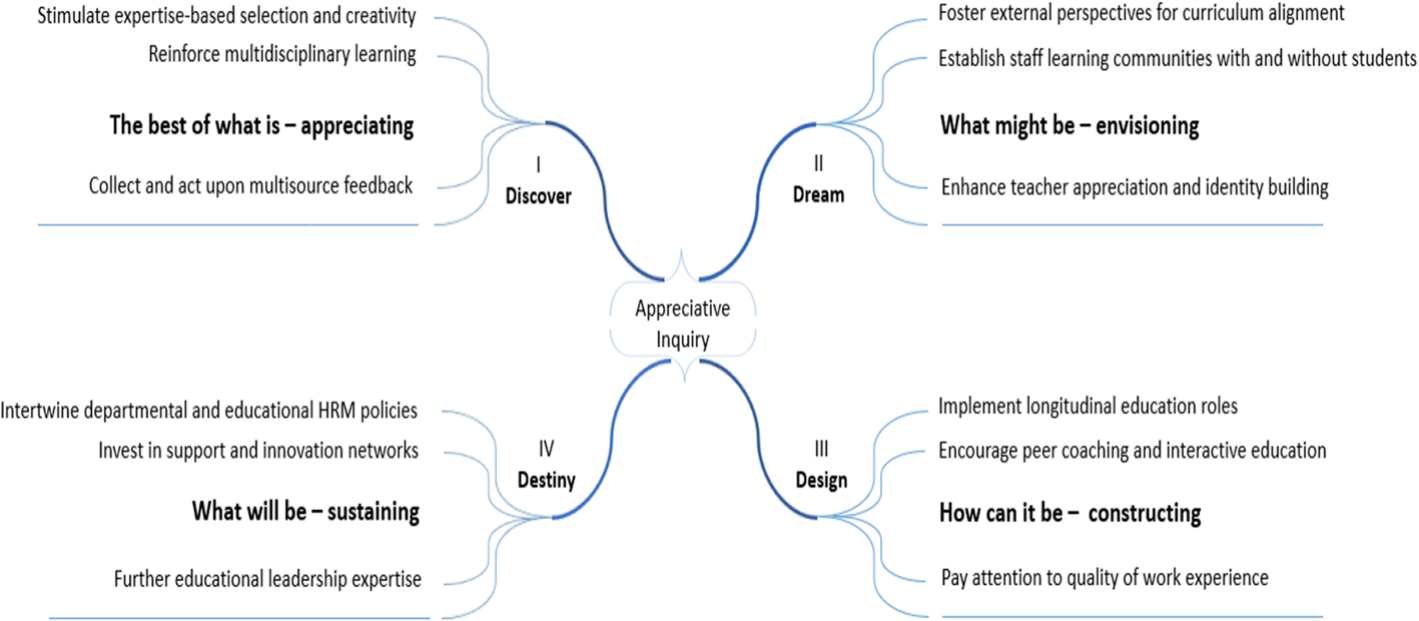
Overview of themes structured under the four phases of appreciative inquiry. The figure presents four interconnected phases of appreciative inquiry: energising and strength-based experiences (I-Discover) form foundations of the envisioned positive future (II-Dream). The vision can be operationalised through aligned actions (III-Design), while organisational strategies and policies support and sustain improvement actions in the longer run (IV-Destiny)

Based upon the qualitative analysis, twelve themes were identified which are structured under the four phases of appreciative inquiry: (1) *Discover*: stimulating expertise based selection and creativity, reinforce multidisciplinary learning, collect and act upon multisource feedback, (2) *Dream*: foster external perspectives for curriculum alignment, establish staff learning communities with and without students, enhance teacher appreciation and identity building, (3) *Design*: Implement longitudinal education roles, encourage peer coaching and interactive education, pay attention to quality of work experience, and (4) *Destiny*: intertwine departmental and educational HRM policies, invest in support and innovation networks, further educational leadership expertise (see Fig. 2).

It should be noted that themes described under the Dream and Design phases might already be present (to a limited degree) in the current situation, yet interviewees reported that these aspects should receive more emphasis to enhance the quality culture. Quotes of interviewees, included after each theme description, illustrate the findings. The quotes by bachelor coordinators and directors of education are presented as ‘PC^n’^, which stands for programme coordinator, number 1–6 (randomly assigned for pseudonymisation purposes), whereas course coordinators are referred to as CC^n^, and the cases are randomly coded as P1, P2, and P3.

### Discovering the best of what is: appreciating

#### Stimulating expertise-based selection and creativity

Interviewees explained that the benefits reaped from working in course planning groups build on expertise-based selection and room for staff to use this expertise in creative ways. A main area of leadership attention focuses on safeguarding that ‘the right people are in the right place’ to cover and deliver the content of the programme. An urge of staff to be involved in education that matches their professional interest was considered key to their motivation to engage in educational development. In addition, a sharing of interests and educational values served as oil for the motor of educational development. Leaders can encourage staff by letting them lead in their own competency domains:

Linked to that [course theme], I started searching for the relevant people; those who were… yeah… very enthusiastic about it. I really noticed they were keen to contribute and were creative. The course is genuinely constructed and advanced based on their inner passion […]. Yes, it strongly connects to their core expertise” **(P3, CC**^**1**^**).**

I put a lot of effort into inviting people to deepen their talents and deploy their passion. I try to offer them a lot of space in areas they feel challenged and competent in […]. They are in the lead… *they* are in the lead” **(P2, CC**^**2**^**).**

#### Reinforcing multidisciplinary team learning

Multidisciplinary teacher teams were seen as an important asset of education at FHML. According to respondents, collaboration and knowledge exchange within diversely composed planning groups (with senior and junior staff, multiple departments, and both genders represented) provided levers for team learning*.* The interviewees indicated that they particularly appreciated reflection with team members on development opportunities. As leaders, they also tried to optimise contributions of team members by sharing responsibilities, connecting different points of view, and fortifying joint efforts:

Then you keep on developing… Just by asking questions […], by engaging in this reflection cycle with the three of us and asking everyone ‘what did you think of it, and how would you like to see it changed’? Then I offer them the leeway to do it that way and we can discuss afterwards, like: ‘well, did it improve or not?’ **(P1, CC**^**4**^**).**

I explicitly do not act as a hierarchical leader. I always ask members of the planning group for their input. It is something that is achieved through joint effort, also with a joint awareness that we must execute it in a good way. I am someone who shares responsibilities, someone who wants to connect people and engage them **(P2, CC**^**1**^**).**.

#### Collecting and acting upon multisource feedback

A multitude of information sources is available through which coordinators collect feedback on educational quality (e.g. student review panels, tutors, lecturers, course evaluation surveys, accreditation committees, national surveys, etc.). Most coordinators expressed their appreciation for narrative and just-in-time feedback as opposed to quantitative evaluations. Additionally, participants emphasised the importance of conducting executive roles themselves. They felt that direct contact with students allowed them to receive richer information and feed forward. By being visible and approachable, coordinators collected informal feedback first-hand and were able to act on and react to evaluations more rapidly.

There are numerous points of view, ranging from accreditation panels to staff members, from people in management positions to students. That is important: to bring these different perspectives together and try to at least maintain and preferably improve education continuously **(P1, PC**^**6**^**).**

The connecting element is often the autonomous nervous system. That was not sufficiently recognised by students.[…] You do not notice that yourself, until you meet them and ask for feedback. ‘This is what we intended; did it land this way?’ ‘No, we didn’t get that’. Well, then you do something about it **(P3, CC**^**3**^**).**

### Unveiling dreams: envisioning what might be

#### Foster external perspectives for curriculum alignment

When asked how they envisaged the ideal situation, respondents referred to several present ‘tensions’. One of these tensions concerned having an internal orientation (e.g. focusing on one’s own course) versus an external orientation (taking in a broader perspective to improve alignment and quality of the whole programme). Moreover, the increased attention to an external perspective also related to flexibility and anticipating on dynamics outside the programme; to align with subsequent master programmes, the labour market and broader developments in academia and the society at large:

That you know the content of all other courses… You should also be ready to change things within your own course to create that alignment […]. Because course coordinators often do what they think is right, but that does not have to be aligned with what was already there or with what is happening in other courses **(P3, CC**^**6**^**).**

You should have a way to… we have student evaluations…but what societal and research developments should we connect to and how do we translate these into course improvements? The reflex is to directly go from the evaluation to… perhaps you should build that [external perspective] into the course evaluation **(P1, CC**^**2**^**).**

#### Establish staff learning communities with and without students

A second tension experienced by the staff members we interviewed related to a focus on educational organisation and process aspects versus more content-based discussions on how to improve the programme. Building on their appreciation for peer collaboration, respondents envisioned the establishment of learning communities in which staff regularly meets to discuss personal experiences, challenges and exchange expertise. A staff dream was to also extrapolate the learning that already took place by the cooperation with students in planning groups to a broader level. By means of open and communication with students, staff-student learning communities could be created. The quotes below refer to both these communities:

It’s important that course coordinators see each other on a regular basis. That they tell each other about challenges they face. What goes well and what could be improved. That we learn from each other. That is very useful I believe […] That is making use of the expertise of the [course coordinator] group **(P3, CC**^**1**^**).**

You could very well ask a student: ‘Which teachers perform exceptionally well and why are these people exceptionally good?’ ‘What makes them better than someone else who is average or below average?’ […] We do take note of it, say ‘great’, but leave it at that.[…] We never ask, ‘how do you actually do that?’ **(P2, CC**.^**4**^**).**

#### Enhance teacher appreciation and identity building

A third ‘vision on what might be’ relates to a tension in valuing research, healthcare, and educational roles. According to respondents, the appreciation for conducting teaching roles could be amplified to come to par with the appreciation of achievements in research. Interviewees verbalised that offering career tracks in education (and giving education an equal status to research) could endorse the teacher identity. Furthermore, respondents also related identity building to a sense of staff to contribute to and being part of a greater whole. The sharing of study programme successes could play a role in this:

That people can make a career in education from the start. Often it is like: ‘then you aren’t good at research or something must have failed […]’. That feeling is still present. I think you can only get this right by giving education and research the same status **(P2, PC**^**2**^**).**

I think the sharing of successes. That you say, ‘this is what we have achieved, in this way’. Yes, that would be a good thing. Rather than saying ‘this is what I have achieved with course […]’, you say ‘this is what we have achieved as a bachelor **(P3, CC**^**6**^**).**

### Designing how it can be: constructing

#### Implement longitudinal education roles

The envisioned themes for further development are related to follow-up actions in the ‘design’ phase. Respondents indicated that fostering external perspectives for curriculum alignment could be put into practice by implementing longitudinal (or recurring) education roles. An example provided concerned the role of mentor. In this role, staff guide students during their study career which respondents deem valuable since they can follow and contribute to the student’s development and at the same time gain in-depth knowledge of the programme. By creating a pool of teachers that conduct the role of tutor, trainer or planning group member for multiple years, the identification and support of the programme’s mission can be strengthened as well. At the same time, regular job rotation (within such a fixed pool of staff in executive roles) was suggested to stimulate innovation:

You receive a lot more feedback on the curriculum from students than you normally do… The students tell us ‘this is what we faced’ or ‘that really was an excellent course’. I am not in that course… As a mentor, you learn way more about how the curriculum is actually constructed **(P3, PC**^**3**^**).**

We have colleagues that also perform care tasks and have a background in clinical education. They can play an important part in skills trainings. We have the in-house expertise, but since roles are scattered it is difficult to secure sufficient trainers each year.[…] [In the planning group] we should perhaps move to a system with annual rotations, to exchange knowledge and expertise, but at the same time maintain the dynamics to innovate **(P1, CC**^**6**^**).**

#### Encourage peer coaching and interactive education

The vision of establishing staff (-student) learning communities links to opportunities for peer coaching. For instance, respondents explained that new staff members could be guided by senior staff or ‘buddies’ who provide tips and act as a coach. Also in the context of courses, opportunities were identified to pair senior with junior staff in education activities. The interviewees further reported that establishing a learning community also depended on the creation of learning situations where staff and students benefit from small-scale interactive education (e.g. in small research projects). An increased interaction can be reinforced by longitudinal roles (the previous theme). Staff illustrated that opportunities for interaction with students formed an important motivator for them to teach:

I can help this new person with tips. Like: ‘I wouldn’t do that’ or ‘you should pay attention to that’. Try to transfer my expertise. Every tutor approaches the task in his or her own way. There isn’t one absolute, good way, but you can exchange tricks and … try to coach someone else **(P2, CC**^**5**^**).**

There, education and research are closely linked, and we are thinking as a sort of mini team: ‘how could we do this?’ Then it is not about appreciation, but I get satisfaction out of the fact that I can do nice things […]. You suddenly see the student thinking: ‘I know what it’s about, great, and I can participate’! Then, education is very real **(P3, CC**^**4**^**).**

#### Pay attention to quality of work experience

The vision of enhancing teacher appreciation and identity building links to providing attention to the quality of work experience. Interviewees explained that leadership attention to the individual situation of staff forms a keystone to uphold the quality of work experience: some interviewed leaders already did this by taking into account talent development, stimulating learning on the job, and trying to prevent that teaching demands exceeded the manageable workload. This required that leaders invest in personal approaches to maintain connections with the teaching staff in executive roles:

An element that seems to be neglected in the entire policy… *seems* to be neglected, is the quality of work and work experience […] The entire subject of educational quality isn’t seen in terms of human qualities and the quality of their work […]. The work they do should also produce quality for themselves so to say **(P1, CC**^**1**^**).**

That [conversation with individual teachers] resulted from the workload discussion… what is it that really matters to the individual teacher, what does he run into and what would he want in his teaching career? […] I notice that teachers highly appreciate it when you sit down and talk with them about their individual situation **(P1, PC**^**4**^**)**.

### Fostering a sustainable destiny: what will be

#### Intertwine departmental and educational HRM policies

Respondents explained that human resources management (HRM) policies are important to sustain attention to individual, team and collective (study programme) development***.*** Interviewees mentioned that, to this end, recruitment, retention and promotion policies in departments should be intertwined with the faculty’s strategic aims of education. Departments and programmes require leverage to link teaching, research and care tasks tailored to the capacities and ambitions of staff. Another important prerequisite to sustain development is that resources (budget and time) are protected for teachers and teacher teams to further education. Some respondents indicated that their department’s policy to appoint several staff members that are dedicated 100% to teaching (these staff members hold a position fully based on their involvement in education), contributed to their dedication to grow:

‘What suits you now and what is your zone of proximal development?’ […] ‘What are your opportunities for development and what constitutes your challenge?’ […] perhaps also alter periods; [Make sure] that 50/50 [research/education] is dispersed over multiple years, because sometimes there are research projects that need a lot of attention, but you still have to teach 50% **(P1, CC**^**2**^**).**

I now have a full teaching allocation which gives me the chance to fully develop […] I also think [they should] give space to people who are very committed to education and reward these people. That motivates. That [teacher] prize also motivated me to perform even better **(P3, CC**^**3**^**)**.

#### Invest in support and innovation networks

Interviewees mentioned that embedding innovation networks would allow educational leaders to stimulate innovation. Participants for instance pointed to trying out new educational approaches on a small scale in pilot projects which could later be transferred to a larger scale. Teacher network events were mentioned as supportive to peer learning. Moreover, the teaching coordinators stated that they profited from their investments in constructing an (informal) network with both academic and support staff. To facilitate networking, coordinators reflected that they would benefit from a physical proximity to teaching and support staff, which is a challenge in large programmes with staff from many different departments involved:

By prototyping and trying things out you make a better version. We collectively discover what works and what does not […]. A couple of times it did not work, but, still, we got a lot of information out of it. Next time, you come up with something else that *does* work. That is fun **(P2, PC**^**1**^**).**

That you take a moment and start looking: ‘where lies the expertise and who can you ask for that?’ […] That could be people from block support […] or people from the planning department […] That you know who are working for you and who are performing things for you, the practical training and lab supervisors, that you know who they are **(P3, PC**^**3**^**)**.

#### Further educational leadership expertise

The interviewees reflected that educational leadership training is key to sustain good practices but also to continuously improve in their own role as leaders. The respondents explained that they did not necessarily gain their leadership positions because of their educational expertise: experience in education, job rotations, and commitment to educating future professionals set paths to leadership positions as well. According to respondents, the sustainability of future educational innovations and improvement actions would benefit from increased attention to feedback on educational leadership on different levels, through leadership development tracks and learning by means of co-leadership:

A lot of research is done, and many new ideas are generated by you [department of educational research], but also outside this university. I would appreciate it if more attention would be paid to that. A refresher course for coordinators: ‘these are new developments in the field, consider if you can use that’ **(P3, CC**^**2**^**)**.

Leadership development does not receive the attention it deserves […] I believe that, across the board, more attention could be paid to that. Nobody ever told me how to lead such a planning group. What is expected, and if you perform well in that sense. They do review the course, but they do not evaluate my functioning within the course **(P2, CC**^**1**^**)**.

## Discussion

This study sought to increase insights into how a quality culture can be enhanced according to the experiences and perspectives of educational leaders. The findings highlight that peer learning in multidisciplinary teams and communities as well as attention to continuous professional development form the core of a quality culture. Leaders can play a key role in these spheres of capacity building by initiating learning across teacher networks, and providing room to staff to use their expertise while gradually increasing their responsibilities in education. Educational leaders indicated that attention to quality of work experiences, appreciating and rewarding staff teaching performance, and stimulating room for creativity, nurture quality culture conditions. Overall, the experiences and perspectives of interviewees strongly relate to human interrelations- and human resources as drivers of the organisational quality culture. An increased fit between leadership practices and preferences for educational and organisational development, HRM strategies, support and innovation networks, and leadership training programmes is required to sustain a quality culture in HPE in the long run.

Typical strengths and visions for improvement found in our case study link to investments made in integrated, multidisciplinary courses and close staff, and staff-student interactions. Our findings furthermore resonate with research on conditions for positive teacher team performance. I.e. these conditions can be nurtured by stimulating co-creation, sharing responsibilities and decision making, and through the enhancement of psychological safety in teams by educational leaders (Meeuwissen et al. 2019, 2020; Koeslag-Kreunen et al. 2018). Leaders of educational programmes often cannot influence the broader organisational HRM policies, but if leadership efforts and HRM practices are not fully aligned (e.g. in staff recruitment and promotion policies), opportunities for HRM and leadership to act in synergy are not used to an optimal degree (Leroy et al. 2018). Hence, strategic investments, incentives and accounting for the professional development of staff are needed to support the educational mission (Love et al. 2018; Engbers et al. 2017). Our study also underscores that a lot is still to be gained from levelling appreciation of education roles to roles in research and healthcare as this could allow for a more swift merger of professional identities (Hall 2005; Thomas et al. 2020).

It should be acknowledged that the curriculum integration approaches, longitudinal education roles, alternative reward structures and other forms of governance proposed by interviewees might form only part of the way to advance quality cultures in HPE. As organisations regularly change strategies and processes to adapt to changing circumstances, it can be argued that leadership, relationships, ownership and trust are at the heart of a quality culture, rather than strategies and systems (Kezar 2004). Agreements between educational leaders and academics that precede decision making, based on a reached state of shared educational principles, norms and values, could aid to adopt a quality culture and go beyond implementing quality strategies (Stensaker and Vabø 2013). Four main characteristics attributed to successful leadership are of the essence in this respect. Leaders can (1) articulate inspiring visions for the future, (2) stimulate new ways of thinking, (3) attend to ‘follower’s’ needs and concerns, and (4) act as a role model (Siangchokyoo et al. 2020). For academics to become involved in quality culture enhancement, however, non-leader-centered approaches are also more required. Such approaches are characterised by interaction, a creation of trust, listening, a people focus, and a style of ‘management by walking around’ (Knight and Trowler 2000). Hence, non-leader-centered approaches do justice to the dependency of HEIs on individual staff contributions and their functioning in teams. In this study, we found multiple examples of distributed (but coordinated) leadership (Mehra et al. 2006). Such approaches reflect a balanced quality culture that highlights opportunities to reach organisational goals, while taking individual staff aspirations into consideration to push or pull goals and quality improvement work into the desired direction.

Our findings underscore the importance of collective workplace learning and communities of practice (e.g. Elmberger et al. 2019; Cruess et al. 2018). The good practices reported upon by interviewees surfaced where networking and a sharing of ideas were fostered. Leaders reported to be strongly embedded in such networks, which they also use to their benefit to create support for change. The finding that leadership is considered to influence quality culture enhancement indirectly, through networks and influencing values, is in line with studies that advocate a human relation orientation (Bendermacher et al. 2019; Blouin et al. 2019). Their indirect influence explains why leaders may “resemble the abominable snowman whose footprints are everywhere, but who is nowhere to be seen” (Bennis and Nanus 1997, p.4). Our results imply that HPE institutions benefit from those (leaders) who shift from a focus on isolated, role based actions of individuals to the influences of contexts on processes, behaviours, and outcomes (Lichtenstein et al. 2006). Leaders that are able to adopt such a contextual perspective might be more inclined to recognise the importance of crossing boundaries of departments and subcultures in order to nurture a shared educational quality culture. These often are experienced staff members that possess the competencies to balance tensions (e.g. relating to internal control versus external dynamic demands) and use these tensions to drive improvement (Hill and Stephens 2005).

This study adds to the field of quality culture and leadership research by including views from leaders working on different levels. In addition to their focus on human relations, leaders’ experiences on quality culture enhancement encompassed management aspects and organisational structure issues. Rules, policies, procedures, and accountability, are also needed for a quality culture to take root. The balancing of structures and systems with staff values requires coalition building, negotiation and mediating for resources (Bolman and Deal 2003). We noticed that in the interviews, especially bachelor coordinators and directors of education mentioned the influential role of management structures, strategic issues and systems for accountability. The same holds for visions to enhance curriculum alignment. Course coordinators appeared to focus more on improvement of content and team learning aspects. These differences can logically be explained by role characteristics which range from more team-focused behaviours on course level to political and strategic behaviour on the overarching domain level. As competencies to use different frames of leadership add to the organisation’s adaptability, it is worthwhile to offer training in domains HPE leaders are not that accustomed to (Sasnet and Clay 2008; Bearman et al. 2018). This is not to say that human relations approaches should be abandoned (on the contrary). However, training that allows (future) leaders to consider the wider context can fuel opportunities to eventually change these contexts, not only the individuals within it. In this sense, the legacy of HPE leaders for quality culture enhancement also entails the imprint they make at the end of their tenure by leaving many good leaders behind who can go even further (Fullan 2005).

In addition to the gaps our study addresses, the added value of our research is to be found in the application of appreciate inquiry. So far, the use of this approach in HPE research is limited (Sandars and Murdoch-Eaton 2017). Several participants expressed to value the focus on the positive and energising aspects our research method encompassed. The combination of appreciative inquiry with the use of vignettes aided to stimulate dialogue and reflection, which in a natural way led to the generation of insights and new ideas.

### Limitations

The fact that this research is conducted in the setting of three study programmes offered within one organisation should be taken into account in the interpretation of the results. Our results are not necessarily generalisable or transferable to other populations or settings due to the relatively small sample size. The insights we gained are very relevant for hypothesis generation and further research on quality culture enhancement in HPE, but similar research in other organisations is required to further underpin our conclusions. A second limitation concerns the fact that our results represent self-reports and perceptions of educational leaders. In-depth questions on how interviewees experienced their own situation, and probes to illustrate these with examples and anticipated effects of organisational developments, aided to provide a truthful picture. Nevertheless, a triangulation on the experiences and perspectives of leaders and their qualities by students and/or other teaching staff members are required to gain a holistic perspective on quality culture enhancement and the specific contributions of leaders herein. A quality culture stresses the importance of a broad stakeholder feedback and involvement in order for improvement and innovation to thrive.

### Future directions

Some of the main challenges of HPE institutions today lay in crafting curriculum alignment, fostering professional development of teaching staff and anticipating on external demands to continuously evolve. The results of this study reveal that these challenges should be addressed by placing human resources and interrelations central as the institution’s greatest assets for quality culture enhancement. Educational leadership alone will not be sufficient to advance the quality culture. However, by focussing on networking, communication, and coalition building, leaders can stimulate the quality of study programmes to exceed the sum of their individual parts. Educational research and faculty development training that focus on learning leadership in context (‘on the job’), with room for reflection on personal views, experiences, and competencies, are needed to remain fit for the future.
